# Somatic cancer mutations in the MLL3-SET domain alter the catalytic properties of the enzyme

**DOI:** 10.1186/s13148-015-0075-3

**Published:** 2015-03-28

**Authors:** Sara Weirich, Srikanth Kudithipudi, Ina Kycia, Albert Jeltsch

**Affiliations:** Institute of Biochemistry, Faculty of Chemistry, Stuttgart University, Pfaffenwaldring 55, Stuttgart, 70569 Germany

## Abstract

**Background:**

Somatic mutations in epigenetic enzymes are frequently found in cancer tissues. The MLL3 H3K4-specific protein lysine monomethyltransferase is an important epigenetic enzyme, and it is among the most recurrently mutated enzymes in cancers. MLL3 mainly introduces H3K4me1 at enhancers.

**Results:**

We investigated the enzymatic properties of MLL3 variants that carry somatic cancer mutations. Asn4848 is located at the cofactor binding sites, and the N4848S exchange renders the enzyme inactive. Tyr4884 is part of an aromatic pocket at the active center of the enzyme, and Y4884C converts MLL3 from a monomethyltransferase with substrate preference for H3K4me0 to a trimethyltransferase with H3K4me1 as preferred substrate. Expression of Y4884C leads to aberrant H3K4me3 formation in cells.

**Conclusions:**

Our data show that different somatic cancer mutations of MLL3 affect the enzyme activity in distinct and opposing manner highlighting the importance of experimentally studying the effects of somatic cancer mutations in key regulatory enzymes in order to develop and apply targeted tumor therapy.

**Electronic supplementary material:**

The online version of this article (doi:10.1186/s13148-015-0075-3) contains supplementary material, which is available to authorized users.

## Background

The mixed lineage leukemia (MLL) family of histone lysine methyltransferases consists of several proteins including MLL1-5, SET1a, and SET1b. MLL1 and MLL2 are related to Drosophila Trithorax (Trx), MLL3 and MLL4 related to Drosophila Trithorax related (Trr), and SET1A and SET1B are related to dSet1 [1]. MLL proteins are capable of introducing mono-, di-, and trimethylation of histone H3 at lysine K4. Each methylation state of H3K4 is associated with a distinguished chromatin state, for example, H3K4 monomethylation is majorly located at enhancer elements and H3K4 trimethylation is associated with the promoters of the active genes. Recent work has demonstrated that MLL3/MLL4 function as major H3K4 monomethyltransferases at enhancers [2,3]. MLL proteins function as large complexes that include tryptophan-aspartate repeat protein-5 (WDR5), retinoblastoma-binding protein-5 (RBP5), and absent small homeotic-2-like (ASH2L) as core complex members, which are indispensable for the complete methyltransferases activity, plus variable additional factors [4-7]. The MLL3 (KMT2C) protein is 4,911 amino acids long, and it contains 8 plant homeodomain (PHD) and a suppressor of variegation, enhancer of zeste, trithorax (SET) domain which contains the catalytic center. Knockout of MLL3 in mice led to stunted growth, reduced cell proliferation, and lower fertility [8].

In general, cancer is caused by mutations and epigenetic alterations. These effects overlap when epigenetic factors are mutated, for example, EZH2, DNMT3A, or TET2, which are frequently affected [9,10]. MLL3 is considered as tumor suppressor gene because it is often deleted in myeloid leukemia patients [11], and the targeted inactivation of MLL3 in mice leads to epithelial tumor formation [12]. Correspondingly, recent studies reported reduced MLL3 expression in many breast tumors [13,14], and low expression of MLL3 was correlated with the poor survival rate in the gastric cancer patients as well [15]. In addition, MLL3 is also recurrently mutated in several cancers including glioblastoma, melanoma, pancreatic, and breast cancers, and overall is one of the most frequently mutated PKMTs in cancers [11,16,17].

Ongoing sequencing studies uncovered a large number of somatic mutations in cancer tissues, but it is difficult to discriminate relevant driver mutations from irrelevant so-called passenger mutations [18]. One approach in this direction is to study the effect of the mutations and investigate if critical properties of the protein are affected, as done here with three mutations reported to occur in the catalytic SET domain of MLL3. Two of them led to massive changes of the enzymatic properties *in vitro* and in cells - N4848S abolished the catalytic activity and Y4884C changed the product pattern of the enzyme leading to increased generation of H3K4me2 and me3, while the MLL3 wild-type only deposits H3K4 monomethylation. Our data indicate that somatic mutations in the SET domain of MLL3 alter its catalytic properties indicating that the mutations might contribute to carcinogenesis in a distinct mutation-specific manner.

## Results

### Somatic mutations in the SET domain of MLL3 affect its catalytic activity

**Figure 1:**
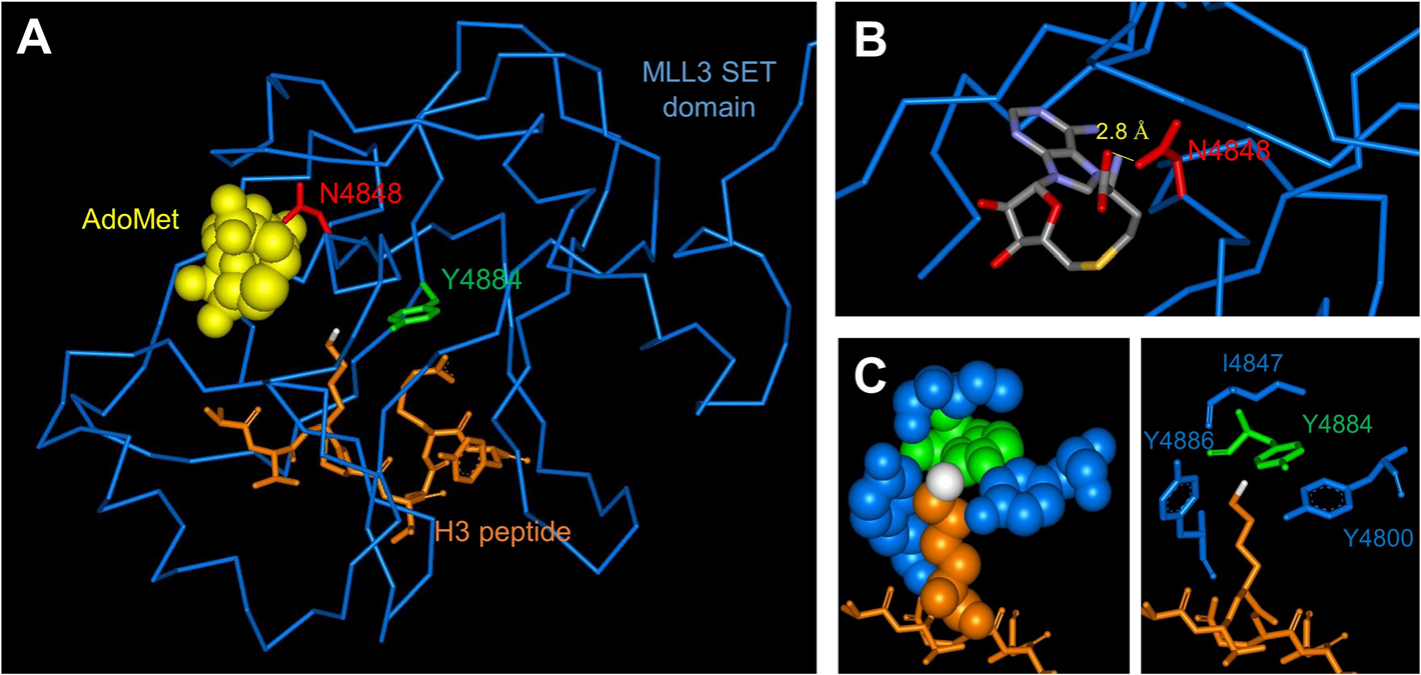
**Structure of the MLL1-SET domain bound to the H3 peptide and cofactor product S-adenosyl-L-homocysteine (AdoHcy) (pdb code 2W5Y).** Note that an MLL3 structure currently is not available. **(A)** The protein is shown as blue ribbon, and the peptide is shown in orange in the stick model with the target nitrogen atom colored white. The residues corresponding to Asn4848 and Tyr4884 are displayed in red and green, respectively, the corresponding alignment of MLL1 and MLL3 is shown in Additional file 1: Figure S4. **(B)** Details of the MLL1-SET structure showing that N3906 (corresponding to N4848) is involved in an H-bond to AdoMet (shown in stick model with coloring by atom type). **(C)** Details of the MLL1-SET structures showing the hydrophobic and aromatic pocket of MLL1 surrounding the target nitrogen atom which consists of Y3942 (corresponding to Y4884, shown in green) and Y4800, I4847, and Y4885 (all designations refer to MLL3, residues shown in blue).

**Figure 2:**
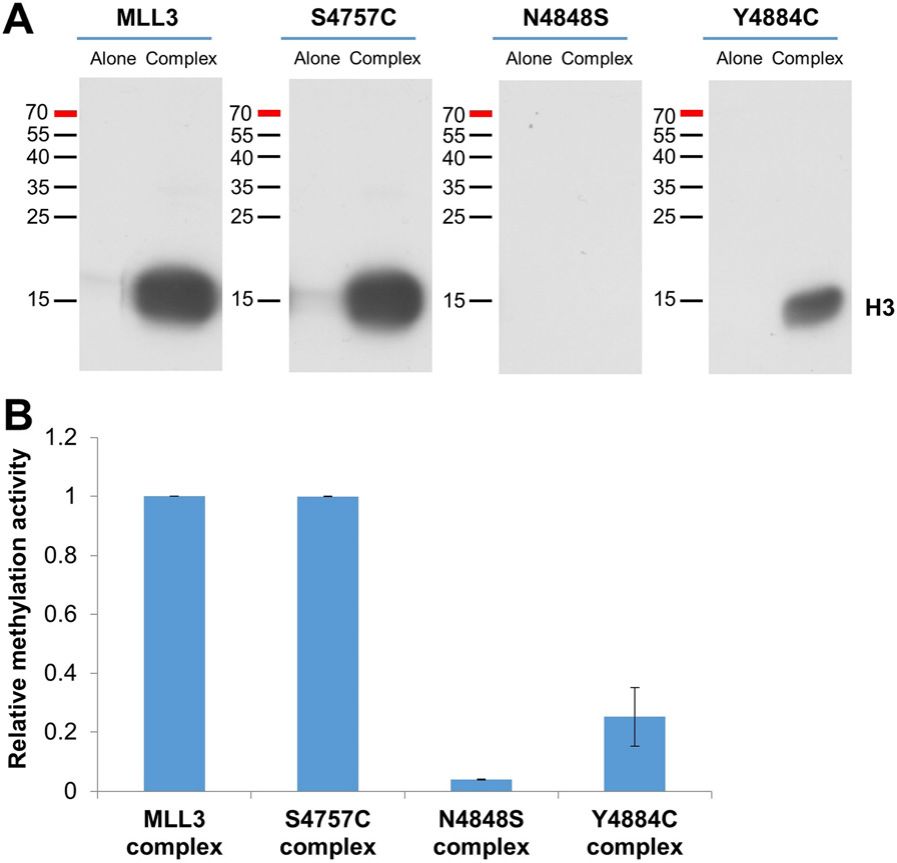
**Activity of MLL3 protein variants.** Recombinant histone H3 protein was methylated with radioactively labeled AdoMet by MLL3-SET wild-type and MLL3-SET mutant proteins either alone or in the presence of complex member proteins. **(A)** Example of an autoradiographic image of the SDS polyacrylamide gel. The methylation signal of H3 is indicated. **(B)** Quantitative analysis of the averages of duplicate experiments. The error bars indicate the standard error of the mean.

Using the COSMIC database [19], we searched for missense mutations in the SET domain of MLL3 (4,771 to 4,911) and selected three mutations (S4757C, N4848S, and Y4884C) based on their proximity to the peptide or AdoMet binding sites of the enzyme (Figure 1). The mutations were found in cancers of the lung (S4757C), endometrium, central nervous system (N4848S), and large intestine (Y4884C). The SET domain of human MLL3 and the respective mutations were cloned, and the proteins expressed and purified with good yield (Additional file 1: Figure S1A). The methylation activity of the MLL3 variants was tested with and without complex members (WDR5, RBP5, and ASH2L) using recombinant H3 protein as substrate and radioactively labeled AdoMet. The reaction mixture was separated using sodium dodecyl sulfate-polyacrylamide gel electrophoresis (SDS-PAGE) and the transfer of radiolabeled methyl groups to the H3 protein was analyzed by autoradiography. The results showed that the S4757C and Y4884C MLL3 variants exhibit methyltransferase activity but N4848S is inactive. Similar to wild-type MLL3, the two active variants (S4757C and Y4884C) showed stronger methyltransferase activity in the presence of the complex members (Figure 2A). The methylation activity of the S4757C variant was in par with wild-type MLL3, and the activity of Y4884C was fourfold reduced (Figure 2B). We have analyzed the secondary structure composition of the proteins by circular dichroism spectroscopy indicating that the mutant proteins are correctly folded (Additional file 1: Figure S1B).

### Substrate specificity of MLL3 variants with peptide substrates

**Figure 3:**
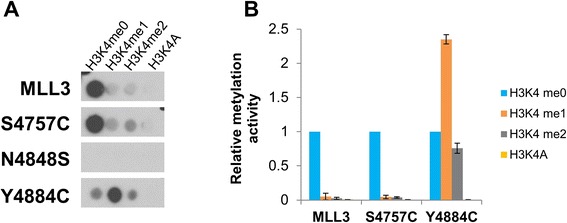
**Substrate specificity of MLL3 protein variants.** H3 (1 to 15) peptide arrays containing H3K4 at different methylation states and a K4A peptide were methylated with MLL3-SET wild-type and mutant proteins in the presence of complex members using radioactively labeled AdoMet. **(A)** Example of an autoradiographic image of the methylated peptide SPOT arrays, peptides with the corresponding lysine variants are indicated. **(B)** Quantitative analysis of the average methylation signals of two independent experiments. The methylation data were normalized to the H3K4me0 methylation signal obtained with the individual MLL3-SET variants. The error bars indicate the standard error of the mean.

To investigate the substrate specificity and product pattern of the MLL3 protein variants, SPOT peptide arrays were synthesized with H3 tail (1 to 15) peptides, which contain either unmethylated H3K4 or different methylated forms of K4. The K4A variant was used as negative control. Peptide arrays were methylated with the MLL3 protein variants in the presence of complex members using radioactively labeled AdoMet (Figure 3). Consistent with the results of the protein methylation assay, the N4848S variant was inactive and both Y4884C and S4757C were active. However, we observed a change in the substrate specificity of the Y4884C variant when compared with wild-type MLL3. Wild-type MLL3 prefers the unmethylated H3K4 peptide as a substrate and no methylation signal was detected with the methylated forms of H3K4 and on the H3K4A, indicating that it could only introduce a single methyl group on H3K4, which is in agreement with literature data [2,3]. Strikingly, Y4884C preferred the H3K4me1 peptide substrate and some methylation activity was even observed on the H3K4me2 substrates illustrating that this variant can transfer up to three methyl groups to the target lysine. At the same time, the variant was less active on the unmethylated H3K4 substrate, indicating a pronounced change in enzymatic properties when compared to the wild-type enzyme.

**Figure 4:**
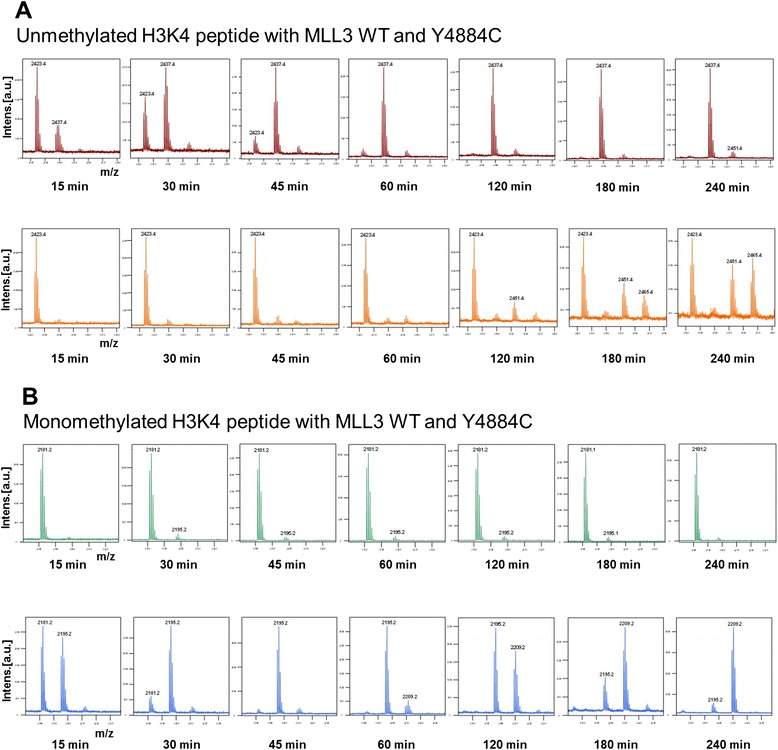
**Substrate specificity and product pattern of MLL3 protein variants.** H3K4 unmethylated (H3; 1 to 19) and monomethylated H3 (1 to 17) peptides were methylated by MLL3-SET wild-type and MLL3-SET Y4884C mutant protein in the presence of complex members using unlabeled AdoMet. The samples were collected at different time points, and methylation was analyzed by mass spectrometry using the relative areas of the corresponding unmethylated and methylated peaks. **(A)** Matrix-assisted laser desorption/ionization (MALDI) spectra of the methylation of the H3K4me0 peptide with MLL3 wild-type (upper panel) and Y4884C (lower panel). The masses of the corresponding peptides are 2,423.4 Da (H3K4me0), 2,437.4 Da (H3K4me1), 2,451.4 Da (H3K4me2), and 2,465.4 Da (H3K4me3). **(B)** MALDI spectra of the methylation of the H3K4me1 peptide with MLL3 wild-type (upper panel) and Y4884C (lower panel). The masses of the corresponding peptides are 2,181.2 Da (H3K4me1), 2,195.2 Da (H3K4me2), and 2,209.2 Da (H3K4me3).

To further confirm the change in substrate methylation state preference and product pattern of the Y4884C mutant, unmethylated and monomethylated H3K4 peptides were incubated in solution with MLL3 wild-type and the variants in the presence of unlabeled AdoMet. The samples were collected at the defined time intervals, and the methylation was analyzed by mass spectrometry. Wild-type MLL3 showed fast monomethylation of the H3K4me0 peptide, followed by weak dimethylation after long incubation times (Figure 4A, upper panel). No methylation was detectable with the H3K4me1 peptide even after the long incubation times (Figure 4B, upper panel) which suggests that the MLL3 wild-type protein is inactive on the H3K4me1 peptide and it is majorly a H3K4 monomethyltransferase. The weak dimethylation of the H3K4me0 peptide likely is due to a processive methylation of bound peptide after the first methylation cycle. In contrast, the Y4884C variant exhibited only weak activity on the H3K4me0 peptide (Figure 4A, lower panel). However, this did not result in an accumulation of monomethylated products indicating that they were rapidly converted into the di- and trimethylated state. Strikingly, Y4884C showed strong methylation activity on the H3K4me1 peptide resulting in the generation of dimethylated and later trimethylated products (Figure 4B, lower panel).

**Figure 5:**
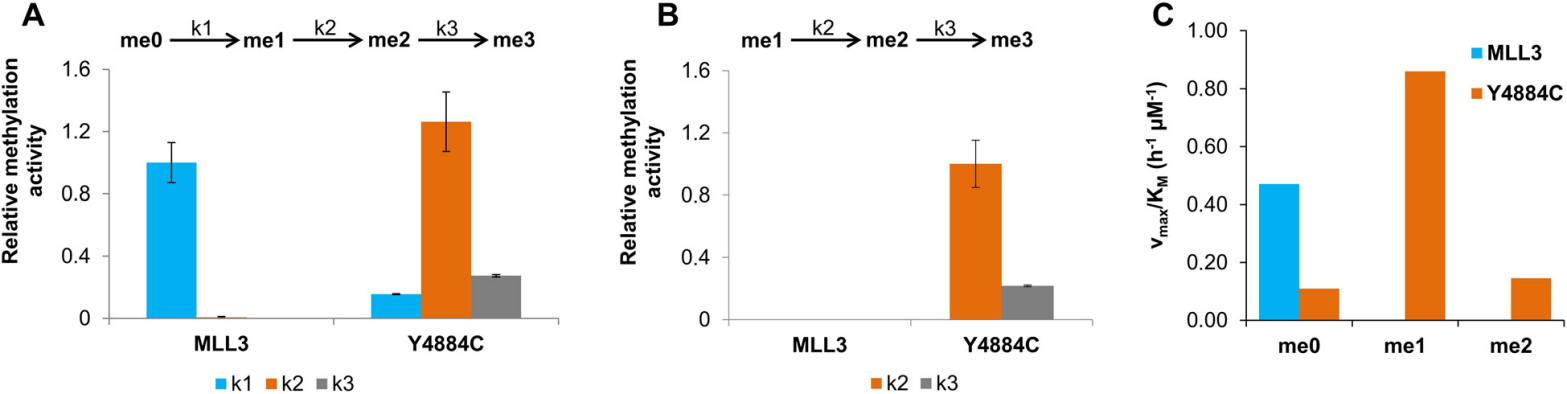
**Summary of the analysis of the results of the peptide methylation experiments.** Panels **(A)** and **(B)** show the average rate constants of MLL3-SET wild-type and MLL3-SET Y4884C variant for the methylation of H3K4me0 **(A)** and H3K4me1 **(B)** substrates determined at a peptide concentration of 10 μM. Error bars indicate the standard error of mean of two independent experiments. Data in panel **(A)** were normalized to *k*_1_ of MLL3-SET, and data in panel **(B)** were normalized to *k*_2_ of Y4884C. Panel **(C)** shows the *v*_max_/*K*_*M*_ values for methylation of the un- (me0), mono- (me1), or dimethylated (me2) peptide substrates by MLL3-SET and Y4884C. These values were determined from methylation kinetics carried using 5, 10, 20, and 40 μM of peptide (Additional file 1: Figure S5).

The methylation data were fitted by numerical integration and least squares fit to a non-processive kinetic model. The H3K4me0 data show that MLL3 wild-type has a high preference for H3K4me0 and conversion rate of me1 to higher methylated forms is almost zero (Figure 5 and Additional file 1: Figure S2). In contrast with the H3K4me0 substrate, the Y4884C mutant showed highest activity on the monomethylated substrate. This conclusion was supported by the methylation of the H3K4me1 substrate which was rapid with the Y4884C variant. The S4757C variant showed similar preferences and activities as the wild-type (data not shown). These results indicate that the Y4884C variant has an altered substrate specificity and product pattern and unlike the other two active variants, Y4884C is a unique H3K4 variant of MLL3 with di- and trimethyltransferase activity.

Next, we determined the rates of peptide methylation of MLL3 and the Y4884C variant using un-, mono-, and dimethylated peptide substrates at variable peptide concentrations of up to 40 μM and fitted the initial rates to the Michaelis-Menten model. While the resulting *K*_*M*_ values were too high to allow a reliable fitting of the individual *K*_*M*_ and *v*_max_ values, the *v*_max_/*K*_*M*_ values, which represent the most valuable parameter to compare enzyme activities, were well defined (Figure 5C). These experiments confirmed that wild-type MLL3 is more active than Y4884C on the unmethylated peptide substrate, but it has almost no activity on mono- or dimethylated substrates. In contrast, the Y4884C variant prefers the monomethylated substrate and it can also methylate the dimethylated substrate.

### Substrate specificity of MLL3 variants with protein substrates

**Figure 6:**
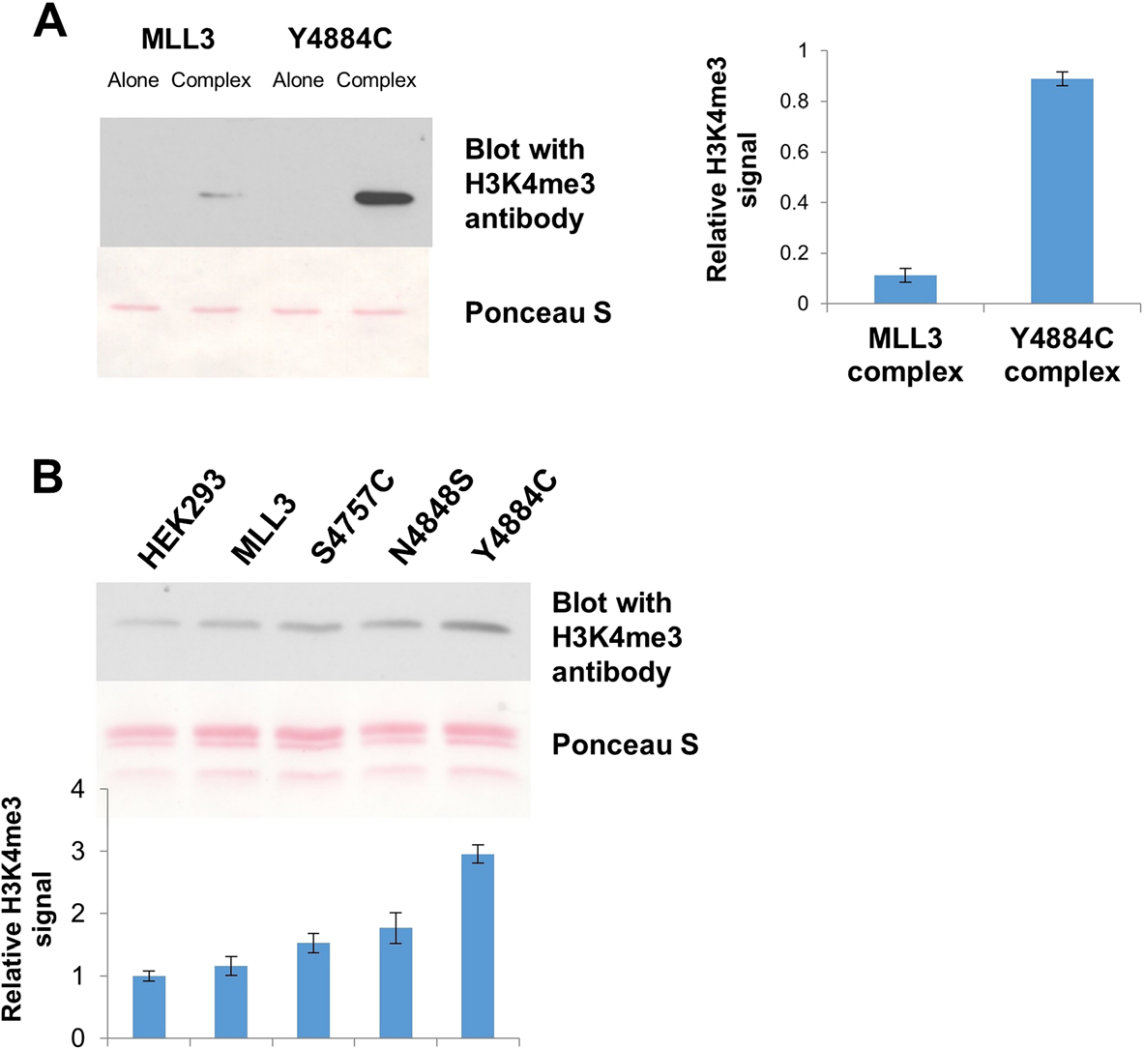
**Product specificity of MLL3-SET wild-type and Y4884C at protein level. (A)** Methylation of recombinant histone H3 protein by MLL3-SET wild-type and Y4884C alone and in the presence of complex members using unlabeled AdoMet as cofactor. After methylation, the proteins were separated by SDS-PAGE, blotted and the methylation was detected using an H3K4 trimethylated antibody (upper panel). The lower panel shows the Ponceau S-stained image of the blot. The bar diagram shows the average methylation signal from two independent experiments. The error bars indicate the standard error of the mean. **(B)** Global histone H3K4me3 methylation analysis from HEK293 cells. The cells were transfected with different MLL3 variant plasmids, histones were isolated, and H3K4me3 methylation was probed by Western blot. The upper image shows the H3K4me3 signal and a Ponceau S stain as loading control. The bar diagram shows the quantification from three experiments. The error bars display the standard error of the mean. The signal obtained from the MLL3 was set to 1, and the other signals were normalized accordingly.

Next, we aimed to test the substrate specificity and product pattern of wild-type MLL3 and the Y4884C at the protein level. Recombinant H3 protein was methylated using the same concentrations of MLL3 wild-type and Y4884C variant with and without complex member proteins in the presence of unlabeled AdoMet. The reaction mixtures were separated on SDS-PAGE, and the methylation was detected with an H3K4me3 antibody. The results show a strong trimethylation signal for the Y4884C but only a very faint signal with the MLL3 wild-type in the presence of complex patterns (Figure 6A). No methylation signal was detected with both the variants in the absence of complex members. This result indicated that the trimethylation activity of the Y4884C variant is strongly increased also with protein substrates. The activity of the S4757C variant was similar to the wild-type in this assay as well (Additional file 1: Figure S3).

To study the effect of the MLL3 variants on global H3K4 trimethylation levels in cells, we transiently expressed the MLL3 wild-type SET domain and each of the variants in HEK293 cells. Histone proteins were purified from these cells, and global H3K4me3 levels were determined with the H3K4me3 antibody. As shown in Figure 6B, the expression of the Y4884C mutant protein resulted in a considerable increase of cellular H3K4me3 levels when compared with the histone proteins isolated from the cells transfected with the other MLL3-SET variants, which is in agreement with the *in vitro* result that Y4884C has a trimethylation activity. Although H3K4me3 levels from the cells expressing the other variants (MLL3 wild-type, S4757C, N4848S) were little higher than those of untransfected cells, these differences were not significant. We conclude that the Y4884C somatic cancer mutant of MLL3 exhibits different enzymatic properties than the wild-type protein and it functions as H3K4 trimethyltransferase both *in vitro* and in cells.

## Discussion

It has recently been reported that the somatic mutations in the SET domain of PKMTs, for example, in EZH2 and NSD2, lead to carcinogenesis by altering the global chromatin methylation levels (review [17]). We show here that the N4848S and Y4884C somatic mutations of MLL3 have remarkable and specific effects on the catalytic properties of the enzyme, because the N4848S exchange renders MLL3 inactive and the Y4884C exchange converts MLL3 from a monomethyltransferase to a di- and trimethyltransferase. These pronounced changes of the catalytic properties of both variants strongly suggest that the mutations have a direct role in carcinogenesis. The lack of effects of the S4757C exchange does not exclude that other properties not covered by our assay may be altered. Alternatively, it may indicate that this is a passenger mutation, which has no role in carcinogenesis.

The loss of methyltransferase activity due to the N4848S mutation is not surprising from a structural point of view, as this residue is located in a catalytically important NHXC motif which is highly conserved in PKMTs [20,21]. N4848 in MLL3 is directly located in the AdoMet binding pocket of MLL3 (Figure 1) and the exchange of asparagine to serine affects a hydrogen bond between the cofactor and the protein, which could explain the loss of activity. This result is in agreement with a tumor suppressor role of MLL3, because many cancers were reported to have either reduced expression of MLL3 or inactive truncated proteins due to frame shift mutations.

The change in substrate preferences and product pattern of the Y4884C mutant can be explained as well, because Y4884 is part of an aromatic active site pocket of MLL3 (Figure 1) and mutations of the aromatic pocket residues have been shown to alter the product pattern of PKMTs [20,22]. The MLL3 Y4884C mutant resembles in its properties the enhancer of zeste homolog 2 (EZH2) mutations at Y641, which is the most frequently mutated residue in this enzyme [23]. EZH2-Y641 mutated proteins show different substrate preference and higher H3K27 trimethylation activity than the EZH2 wild-type both *in vitro* and in the tumor cell lines [24-26]. By this, they strongly influence the expression of polycomb repressive complex 2 (PRC2) target genes [27], and EZH2 inhibitors have been successfully used in tumors with these mutations [27,28]. The MLL3 Y4884C variant could lead to similar changes of the global chromatin regulation.

## Conclusions

We report here the effect of somatic mutations in the catalytic domain of MLL3. Our data show that mutations in the SET domain of MLL3 may lead to tumorigenesis through two converse mechanisms. The N4848S mutation leads to a loss of the catalytic activity of MLL3, which resembles the effect of other loss of function mutations in MLL3, like frame shifts or loss of expression. Such mutations may lead to the loss of H3K4 methylation at target genes and inhibit the expression of tumor suppressor genes. The Y4884C mutation leads to a change in the substrate specificity and product pattern of MLL3. This may result in the deposition of aberrant H3K4 trimethylation at enhancers leading to their conversion to promoters and the expression of oncogenes similarly as observed after knockdown of the Kdm5c H3K4me3 demethylase [29]. Hence, MLL3 inhibitors are promising therapeutic options for cancers containing Y4884C mutations, but they might even be harmful in cancers with N4848S mutations. These data illustrate that individual cancer mutations even in one protein need to be functionally studied in order to develop and apply individual treatments.

## Methods

### Cloning, expression, and purification of proteins and protein variants

MLL3-SET domain (4,734 to 4,911, Uniprot identifier-Q8NEZ4-1) was amplified from the cDNA prepared from HEK293 cells and cloned into pGEX-6p2 vector as GST fusion protein. For mammalian expression, coding sequence of MLL3-SET domain was similarly amplified and cloned into pEYFP-C1 vector (Clontech, Palo Alto, CA, USA). The corresponding mutations in the MLL3-SET domain were generated using megaprimer PCR protocol. For expression, *Escherichia coli* BL21-DE3 codon plus (Novagen, Madison, WI, USA) carrying the corresponding plasmid were grown at 37°C until they reached 0.6 to 0.8 OD_600_. Cells were then shifted to 20°C for 10 min and induced overnight with IPTG (1 mM). The cells were harvested by centrifugation (5,000 *g*). Protein purification was conducted as described before [30]. For eukaryotic expression, the MLL3-SET domain and corresponding mutant plasmids were transfected in HEK293 cells using FuGENE HD (Promega, Madison, WI, USA). The cells were harvested 3 days after transfection. Histones were isolated by the acid extraction method as described previously [31]. The WDR5, RBP5, and ASH2L proteins were expressed and purified as described [32].

### Peptide methylation assays

Peptide arrays on cellulose membranes were synthesized using SPOT method and methylated as described [33]. Peptides used for the in solution experiments were commercially purchased from Intavis AG (Köln, Germany). Peptide arrays were washed for 5 min in the methylation buffer (50 mM Tris-HCl (pH 8.0), 200 mM NaCl, 5 mM MgCl_2_, and 3 mM DTT). The arrays were then incubated for 60 min at room temperature in methylation buffer containing 50 nM MLL3-SET variants and 0.76 μM radioactively labeled AdoMet (PerkinElmer, Waltham, MA, USA) and treated as described previously [30,34]. In solution peptide, methylation was performed by incubating the respective peptide in the methylation buffer (50 mM Tris-HCl (pH 8.0), 200 mM NaCl, 5 mM MgCl_2_, and 3 mM DTT) supplemented with 50 nM of MLL3-SET variant protein and unlabeled AdoMet (1 mM). If not otherwise indicated, 10 μM peptide was used. The reaction was carried out at 25°C temperature. Samples were collected from the reaction tube at indicated time points, and the reactions were stopped by diluting 1 μl of the reaction mixture 9 μl 0.1% TFA, and the methylation level of the peptides were analyzed by mass spectrometry as described [35].

### Histone protein methylation assays

The H3 protein was incubated with MLL3-SET in methylation buffer in the presence of either radioactively labeled or unlabeled AdoMet for 3 h at 25°C. The reactions were stopped by heating the samples in the SDS loading buffer at 95°C for 5 min. Then, the proteins were separated on a 16% SDS-PAGE. The methylation signal was detected by autoradiography in the samples methylated with radioactive AdoMet cofactor. For the samples methylated with the unlabeled AdoMet, the methylation signal was detected by Western blot with a modification-specific antibody (Active motif, Cat. # 39159, Lot. # 15808002, which binds to H3K4me3 with very good specificity [36]).

### Circular dichroism analysis

Circular dichroism (CD) measurements were performed using a J-815 circular dichroism spectrophotometer (JASCO Corporation, Tokyo, Japan). MLL3-SET wild-type or the mutant variant proteins (20 μM) were diluted in a buffer containing 10 mM Tris pH 7.5 and 200 mM KCl, and the spectra were collected at room temperature using a 0.1-mm cuvette in a wavelength range between 195 and 240 nm. For each sample, at least 60 scans were collected and averaged. The spectra of mutant proteins were scaled to the wild-type protein to normalize concentration differences and allow better comparison.

## Additional file

Additional file 1: Figure S1. Protein expression and quality. **Figure S2**. Fit of the methylation data shown in Figure 4 to a stepwise methylation model of the corresponding substrate peptide. **Figure S3**. *In vitro* methylation of recombinant H3 protein by MLL3-SET wild-type and S4757C. **Figure S4**. Alignment of the amino acid sequences of MLL1 and MLL3. Figure S5. Rate *vs.* c (peptide) plots obtained from the peptide methylation experiments.
