# Supplementary material for: Somatic cancer mutations in the MLL3-SET domain alter the catalytic properties of the enzyme
Source: Clin Epigenetics. 2015 Mar 28;7(1):36. doi: 10.1186/s13148-015-0075-3 (PMC4379744; doi:10.1186/s13148-015-0075-3)
Supplement: Additional file 1: Figure S1. — Protein expression and quality. Figure S2. Fit of the methylation data shown in Figure 4 to a stepwise methylation model of the corresponding substrate peptide. Figure S3. In vitro methylation of recombinant H3 protein by MLL3-SET wild-type and S4757C. Figure S4. Alignment of the amino acid sequences of MLL1 and MLL3. Figure S5. Rate vs. c (peptide) plots obtained from the peptide methylation experiments. [file 13148_2015_75_MOESM1_ESM.pdf]

Somatic cancer mutations in the MLL3-SET domain alter the catalytic properties of the enzyme

Sara Weirich, Srikanth Kudithipudi, Ina Kycia & Albert Jeltsch*

Supplemental figures und supplemental figure legends

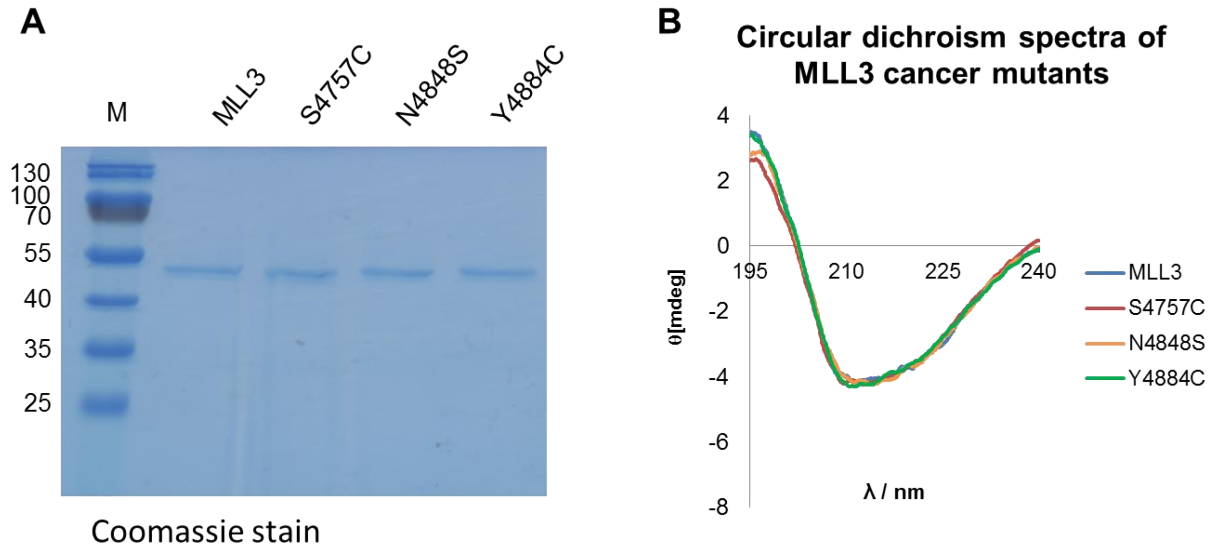

Suppl. Figure 1: Protein expression and quality. A) Coomassie stained gel image of purified MLL3-SET wild type and the S4757C, N4848S and Y4884C variants. B) Circular dichroism (CD) spectra of the purified MLL3-SET wild type and the S4757C, N4848S and Y4884C variants. The spectra of the mutant variants were scaled to the wild type to normalize the differences in the protein concentrations for better comparison.

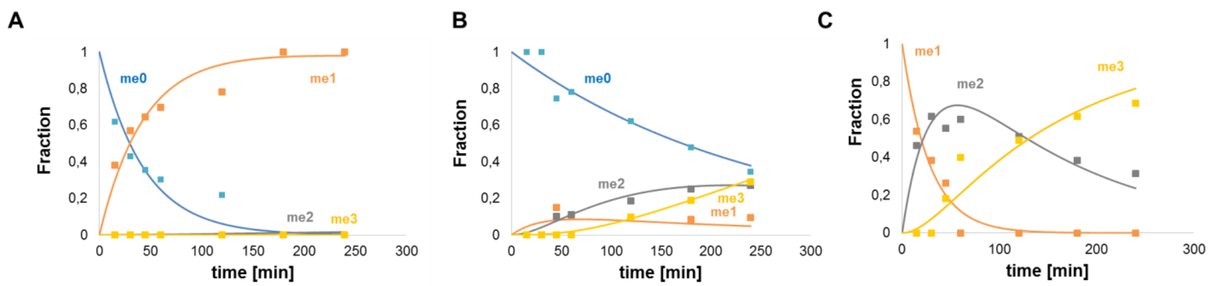

Suppl. Figure 2: Fit of the methylation data shown in Fig. 4 to a stepwise methylation model of the corresponding substrate peptide. The relative rate constants obtained by this fit are shown in Fig. 5. A) Methylation kinetics of H3K4me0 peptide by MLL3-SET wild type (H3K4me0 blue squares, H3K4me1 orange squares, H3K4me2 grey, H3K4me3 yellow squares). The lines show the best fit of the data to the model. B) Methylation kinetics of the H3K4 (1-19) peptide by the MLL3-SET Y4884C variant. The squares and fit are same as described for the panel A. C) Methylation kinetics of the H3K4me1 peptide by the MLL3-SET Y4884C variant. The squares and fit are same as described for the panel A.

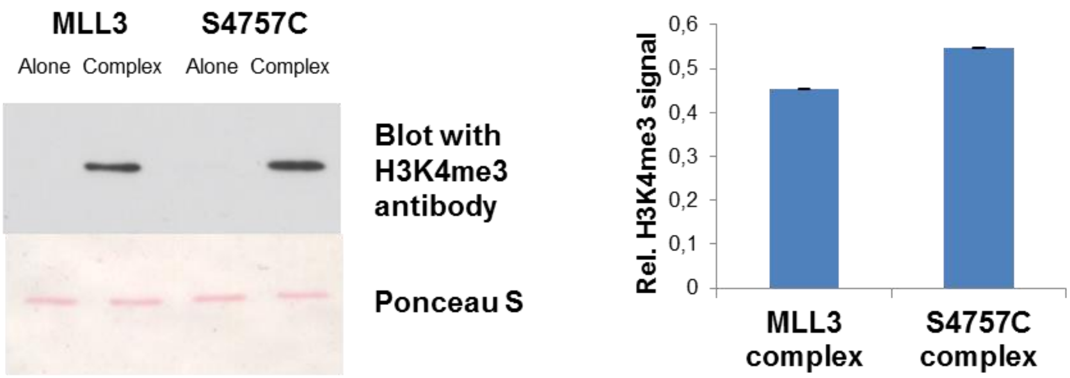

Suppl. Figure 3: Methylation of recombinant H3 protein by MLL3-SET wild type and S4757C. MLL3-SET wild type and S4757C mutant protein as isolated SET domains and in the presence of complex member proteins was assayed using recombinant histone H3 protein as substrate in the presence of unlabeled AdoMet as cofactor. The proteins were separated and methylation activity was detected using H3K4 trimethylated antibody. The upper panel is the immunoblot stained with the H3K4 trimethylated antibody and lower panel is the Ponceau S stained image of H3 proteins used in the assay. The bar diagram represents the quantified methylation activity signal of two experiments. Total methylation signal was set as one and the fraction of methylation signal from wild type and S4757C variant were indicated. Error bars illustrate the standard error of the mean.

```
MLL3 : EASRLYWSTRYANRRCRYLCSIEEKDGRPVFVIRIVEQGHEDLVLSDISPKGVWDKILEPVACVRKKSEMLQLF : 4654
MLL1 : ESNFSSPLMLWLQQEQKRKESITEKKPKKGLVFEISSD--DGFQICAESIEDAWKSLTDKVQEARSNARLKQLS : 3710

MLL3 : PAYLKGEDLFGLTVSAVARIAESLPGVEACENYTFRYGRNPLMELPLAVNPTGCARSEP---KMSAHVKRFVLR : 4725
MLL1 : FAGVNGLRMLGILHDAVVFLIEQLSGAKHCRNYKFRFHK-PEEANEPPLNPHGSARAEVHLRKSAFDMFNFLAS : 3783

MLL3 : PHTLNSTSTSKSFQSTVTGELNAPYSKQFVHSKSSQYRKMKTEWKSNVYLARSRIQGLGLYAARDIEKHTMVIE : 4799
MLL1 : KHRQPPEYNPNDEEEEVQLKSARRATSMDLPMPMRFRHLKKTSKEAVGVYRSPIHGRGLFCKRNIDAGEMVIE : 3857

MLL3 : YIGTIIRNEVANRKEKLYESQNRGVYMFRMDNDHVIDATLTGGPARYINHSCAPNCVAEVVTFERGHKIIISSS : 4873
MLL1 : YAGNVIRSIQTDKREKYYDSKGIGCYMFRIDDSEVVDATMHGNAARFINHSCEPNCYSRVINIDGQKHIVIFAM : 3931

MLL3 : RRIQKGEELCYDYKFDFEDDQHKIPCHCGAVNCRKWMN : 4911
MLL1 : RKIYRGEELTYDYKFPIEDASNKLPCNCGAKKCRKFLN : 3969
```

Suppl. Fig. 4: Alignment of the amino acid sequences of MLL1 and MLL3. The figure shows the C-terminal part of the proteins, which share high similarity. The color code follows Fig. 1. The positions corresponding to MLL3 S4757, N4848 and Y4884 are shaded orange, red and green, respectively. The residues shaded in blue represent the hydrophobic and aromatic pocket of MLL1 and MLL3 (together with Y4884). The yellow shaded E3790 indicates the first residue in the structure of the SET domain of MLL1 (pdb code 2W5Y). Amino acid numbers of MLL1 and MLL3 are indicated on the right.

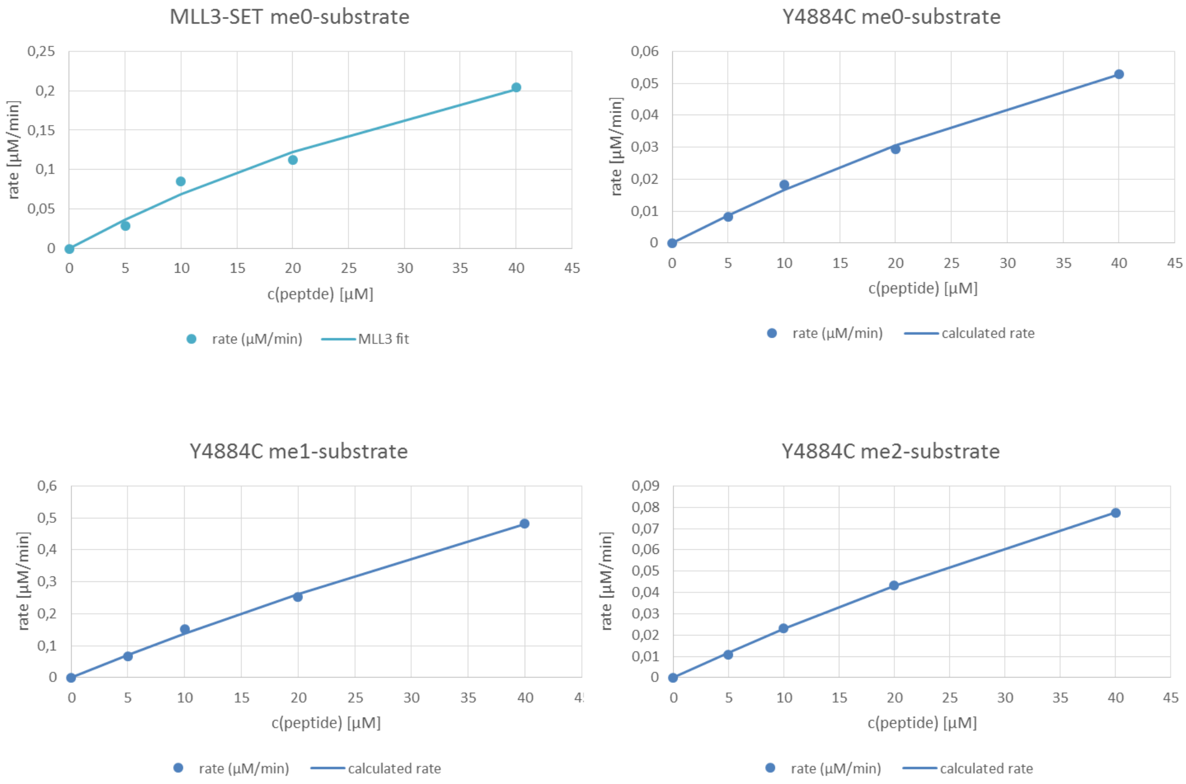

Suppl. Fig. 5: Rate *vs*. c(peptide) plots obtained from the peptide methylation experiments conducted at different substrate concentrations. The fitting did not allow to determine individual K_M and v_{max} values, but v_{max}/K_M was well defined. The corresponding values are shown in Fig. 5C.
